# Supplementary material for: PERSonalised Incentives for Supporting Tobacco cessation (PERSIST) among healthcare employees: a randomised controlled trial protocol
Source: BMJ Open. 2020 Sep 9;10(9):e037799. doi: 10.1136/bmjopen-2020-037799 (PMC7482494; doi:10.1136/bmjopen-2020-037799)
Supplement: Author's manuscript [file bmjopen-2020-037799.draft_revisions.pdf]

BMJ Open is committed to open peer review. As part of this commitment we make the peer review history of every article we publish publicly available.

When an article is published we post the peer reviewers' comments and the authors' responses online. We also post the versions of the paper that were used during peer review. These are the versions that the peer review comments apply to.

The versions of the paper that follow are the versions that were submitted during the peer review process. They are not the versions of record or the final published versions. They should not be cited or distributed as the published version of this manuscript.

BMJ Open is an open access journal and the full, final, typeset and author-corrected version of record of the manuscript is available on our site with no access controls, subscription charges or pay-per-view fees (http://bmjopen.bmj.com).

If you have any questions on BMJ Open's open peer review process please email info.bmjopen@bmj.com

BMJ Open

Personalised incentives for supporting tobacco cessation among health care employees (PERSIST): a randomised controlled trial protocol

Journal:	*BMJ Open*
Manuscript ID	bmjopen-2020-037799
Article Type:	Protocol
Date Submitted by the Author:	17-Feb-2020
Complete List of Authors:	Boderie, Nienke; Erasmus University Medical Center, Department of Public Health<br>van Kippersluis, Hans; Erasmus University Rotterdam Erasmus School of Economics; Tinbergen Institute<br>Ó Ceallaigh, Diarmaid; Erasmus University Rotterdam Erasmus School of Economics<br>Rado, Márta; Erasmus University Medical Centre, Department of Public Health; Erasmus University Medical Centre–Sophia Children's Hospital, Paediatrics<br>Burdorf, Alex; Erasmus University Medical Centre, Department of Public Health<br>Van Lenthe, Frank; Erasmus University Medical Centre, Department of Public Health<br>Been, JV; Erasmus University Medical Centre–Sophia Children's Hospital, Paediatrics, division of Neonatology; Erasmus University Medical Centre, Department of Public Health
Keywords:	PUBLIC HEALTH, PREVENTIVE MEDICINE, SOCIAL MEDICINE, HEALTH ECONOMICS

SCHOLARONE™
Manuscripts

I, the Submitting Author has the right to grant and does grant on behalf of all authors of the Work (as defined in the below author licence), an exclusive licence and/or a non-exclusive licence for contributions from authors who are: i) UK Crown employees; ii) where BMJ has agreed a CC-BY licence shall apply, and/or iii) in accordance with the terms applicable for US Federal Government officers or employees acting as part of their official duties; on a worldwide, perpetual, irrevocable, royalty-free basis to BMJ Publishing Group Ltd ("BMJ") its licensees and where the relevant Journal is co-owned by BMJ to the co-owners of the Journal, to publish the Work in this journal and any other BMJ products and to exploit all rights, as set out in our licence.

The Submitting Author accepts and understands that any supply made under these terms is made by BMJ to the Submitting Author unless you are acting as an employee on behalf of your employer or a postgraduate student of an affiliated institution which is paying any applicable article publishing charge ("APC") for Open Access articles. Where the Submitting Author wishes to make the Work available on an Open Access basis (and intends to pay the relevant APC), the terms of reuse of such Open Access shall be governed by a Creative Commons licence – details of these licences and which Creative Commons licence will apply to this Work are set out in our licence referred to above.

Other than as permitted in any relevant BMJ Author's Self Archiving Policies, I confirm this Work has not been accepted for publication elsewhere, is not being considered for publication elsewhere and does not duplicate material already published. I confirm all authors consent to publication of this Work and authorise the granting of this licence.

For peer review only

BMJ Open

Personalised incentives for supporting tobacco cessation among health care employees (PERSIST): a randomised controlled trial protocol

Nienke W Boderie[1], Hans van Kippersluis[2,3], Diarmaid T O'Ceallaigh[2], Márta K. Radó[1,4], Alex Burdorf[1], Frank J van Lenthe[1], Jasper V Been[1,4]

[1] Department of Public Health, Erasmus MC, University Medical Centre Rotterdam, Rotterdam, The Netherlands

[2] Erasmus School of Economics, Erasmus University Rotterdam, Rotterdam, The Netherlands

[3] Tinbergen Institute, Rotterdam, The Netherlands

[4] Department of Paediatrics, division of Neonatology, Erasmus MC - Sophia Children's Hospital, University Medical Centre Rotterdam, Rotterdam, The Netherlands

Corresponding author:

Dr. Jasper Been

Doctor Molewaterplein 40, 3015 GD Rotterdam

j.been@erasmusmc.nl

+ 31 10 36077

Word count: 4000

For peer review only - http://bmjopen.bmj.com/site/about/guidelines.xhtml

KEY WORDS

Group based training - Financial Incentives - Personalised - Smoking cessation - Validated

ABSTRACT (384 words)

Introduction: Smoking is the primary preventable risk factor for disease and premature mortality. It is highly addictive and the vast majority of cessation attempts are unsuccessful. Incentive-based programmes in the workplace setting seem to be an effective method to reach sustained abstinence. Individualisation of incentives based on personal characteristics yields potential to further increase effectiveness of incentive-based programmes.

Methods and Analysis: A randomised controlled trial among health care workers who signed up for a group-based smoking cessation programme offered at their workplace. The intervention under study is the provision of personalised incentives upon validated sustained smoking cessation at several time points after the smoking-cessation programme. A sample size of 220 participants is required. Participants are randomised 1:1 into intervention (incentives) or control (no incentives). In the intervention arm incentives are provided directly after the smoking-cessation programme and after three, six and twelve months. After 15 months abstinence is validated but no incentive is provided. Incentives are provided as vouchers representing monetary value and provision is conditional on validated abstinence. There are four schemes: (1) Standard: total reward size €350, pay-out scheme: €50, €50, €50 and €200, (2) Descending: total reward size €300, pay-out scheme: €150, €100, €50 and €0., (3) Ascending: total reward size €400, pay-out scheme: €0, €0, €50, €350, and (4) Deposit: total reward size €450, pay-out scheme: €50, €50, €150, €200; participants pay a €100 deposit, which will be returned conditional on abstinence after six months.

Based on the participant's readiness to quit, nicotine dependence and long or short-term reward preference, participants from the intervention arm will be advised which incentive scheme most likely fits their profile. They are however free to deviate from this advice and pick their preferred scheme. Participants in the control arm receive no incentives but abstinence is validated at each time point. The primary end-point is validated abstinence at twelve months. Effectiveness will be determined by intention-to-treat analysis.

Discussion: This trial is unique in its focus on determining the effectiveness of personalised incentives. Findings may further optimise the development of incentive-based programmes to promote smoking cessation in the workplace.

Ethics and dissemination: The Erasmus MC Medical Ethics Committee (MEC-2019-0140) decided that according to the Dutch Human Research Law (WMO) the protocol required no formal ethical approval. All participants will provide informed consent.

Trial registration: Netherlands Trial Register NL7711

STRENGHTS AND LIMITATIONS

- The PERSIST trial has a unique hybrid design with a nudged choice embedded within a randomised trial, and is the first of its kind to provide a personalised advise on the mode of providing incentives which likely best fits individual participant characteristics.
- This trial furthermore addresses a knowledge gap regarding long-term effectiveness of incentive-based strategies beyond the actual intervention period

For peer review only - http://bmjopen.bmj.com/site/about/guidelines.xhtml

- A focus on helping hospital employees quit smoking is essential given their exemplary role in promoting health.
- For logistical reasons participants from the intervention and control arm may attend the same group sessions, potentially leading to spill-over effects.
- Due to the predicted sample size, it is not possible to include an additional intervention arm where incentives are provided but not personalised

For peer review only

For peer review only - http://bmjopen.bmj.com/site/about/guidelines.xhtml

INTRODUCTION

Smoking is the primary preventable risk factor for disease and premature mortality.[1] Smoking is highly addictive and the vast majority of cessation attempts are unsuccessful.[2] In 2017, 38% of smokers in the Netherlands attempted to quit for at least 24 hours.[3] Several methods exist to increase the odds of quitting, such as nicotine replacement therapy,[4] individual behavioural counselling,[5] and group-based smoking cessation programmes.[6] However, the effectiveness of these methods is low.

A promising method to encourage smoking cessation is the provision of financial incentives upon validated abstinence. A recent Cochrane review found high-certainty evidence for increased smoking cessation rates at six or twelve months follow up (RR = 1.49, 95% confidence interval (CI) 1.28 – 1.73).[7] Financial incentives make the desired behaviour - in this case smoking cessation - more attractive by rewarding it. Importantly, financial incentives provide a short term reward for a behavioural change that would normally only provide rewards in the long term. Long term rewards are often valued less compared to short term rewards due to declining perceived values of a delayed reward, so-called delay discounting.[8] Typically, people give disproportionally stronger relative weights to instantaneous rewards, so-called present bias,[9] which gives a strong theoretical argument for providing short-term incentives for smoking cessation.

Incentive-based smoking cessation programmes have previously been implemented at the workplace.[10-12] Recently, the "Continuous Abstinence Through Corporate Healthcare" (CATCH) trial was carried out among 31 Dutch companies, randomising companies to group-based smoking cessation training for smoking employees with or without financial incentives to promote sustained cessation. Among those eligible to receive incentives (up to €350), the proportion of abstinent participants at one year follow-up was significantly higher compared to those assigned to group-based training alone (41% vs 26%).[13]

The effectiveness of incentives in promoting sustained smoking cessation depends on how and when rewards are provided. Traditionally incentives have been tested according to a one-size-fits-all approach, using incentive size as a measure of anticipated effectiveness. A recent US trial comparing four different incentive programs, however, showed that effectiveness is variable among different types and schemes of incentives.[10] A deposit-based scheme, where participants commit their own money and return is conditional on validated abstinence, was unpopular but resulted in a significantly higher proportion of abstinent individuals compared to the reward-based incentives arm.[10] Further analysis revealed associations between personal characteristics and the acceptability and efficacy of incentive programmes.[14] Both readiness-to-quit-smoking and low tendency to discount rewards were associated with increased effectiveness of both reward and deposit based programmes. Personalisation of incentive programmes thus yields the potential to further increase the effectiveness of incentives to promote long term abstinence.

Here we present the study protocol for the "Personalised Incentives for Supporting Tobacco Cessation" (PERSIST) trial. The trial will be conducted among health care employees, for whom smoking cessation is particularly relevant as they may be expected to have an exemplary role. Moreover, in an attempt to work towards a smoke free generation by 2040, the Dutch National Prevention Agreement requires university hospitals to be smoke-free by mid-2020 and all health care facilities by 2025.

The aim of this study is to investigate whether personalised incentives in addition to a group-based smoking cessation programme will increase sustained smoking cessation among participants. This

For peer review only - http://bmjopen.bmj.com/site/about/guidelines.xhtml

unique randomised controlled trial (RCT) attempts to optimise effectiveness of incentive programmes by providing personalised advice while leaving participants in full control to choose the scheme of their choice. Our approach will further provide insight into which incentive schemes participants prefer and if and how they can be nudged into the programme that best fits their personal characteristics.

METHODS AND ANALYSIS

Objectives

The main objective is to evaluate the effectiveness of personalised incentives in combination with group-based training sessions provided in the work environment on sustained smoking abstinence among health care workers compared to group-based training sessions alone.

Trial design

The PERSIST trial is a randomised controlled non-blinded trial with two parallel arms.

Methods

The protocol follows the SPIRIT guidelines for protocol papers.[15]

Study setting

The trial will be implemented at Dutch health care institutions. The trial is primarily implemented at the Erasmus University Medical Centre (Erasmus MC) and is further extended to Franciscus Gasthuis & Vlietland and Ikazia hospital. Inclusion of other health care institutions in the greater Rotterdam area is ongoing.

Trial population and eligibility criteria

Employees are eligible if they are: (1) aged 18 years or over, (2) employed at one of the participating health care institutions, and (3) a daily smoker (i.e. smoking at least one cigarette per day). Exclusion criteria are: (1) only smoking e-cigarettes and (2) not being able to physically attend the group-based training sessions.

Interventions

Eligible participants are individually randomised 1:1 into a control arm, receiving no incentives, and an intervention arm, receiving personalised incentives. Both arms participate in a group-based smoking cessation training provided by SineFuma, a company specialised in supporting smoking cessation (www.rookvrijookjij.nl). Groups consist of eight to 16 participants and the training consists of seven sessions of approximately ninety minutes duration held over a period of eight weeks. Participants in the intervention and control arms participate in the same training sessions for logistical reasons. Group-based training sessions will start as soon as at least eight people have signed up to avoid participants lose motivation due to long waiting times.

Group sessions are provided at locations within each of the participating health care institutions. As part of the training approach, participants jointly quit smoking in the third session. The follow-on sessions then focus on supporting sustained cessation through the first few weeks following the quit attempt. During each session, exhaled carbon monoxide (CO) measurements (see outcomes for more information) are used as a motivator to stay abstinent. The SineFuma group-based training is recognised as an efficient lifestyle intervention by the Dutch Government,[16] and participation costs are reimbursed by basic insurance. As such, the control arm can be considered as usual care. If a

For peer review only - http://bmjopen.bmj.com/site/about/guidelines.xhtml

portion of the costs are at the employee's own expense through the deductible, employers have agreed to pay for the training.

Incentives

The difference between intervention and control conditions lies within the provision of incentives. Participants in the intervention arm who remain continuously abstinent following the joint quit attempt are eligible to receive incentives according to a fixed time schedule. Incentives are provided as vouchers representing monetary value. Vouchers can be spent at online web-shops or on events such as a high tea in a local café or a night at a hotel etc. Vouchers cannot be spent on tobacco products.

In order to personalise incentives, four different incentive schemes are offered, which participants are free to choose from. Across the different schemes, the monetary value of the incentives varies over time, and the potential total financial reward differs. The schemes are as follows (see figure 1):

1. The standard scheme. Analogous to the CATCH trial,[13] vouchers with a monetary value of €50, €50, €50, and €200 are offered upon validated abstinence at t=0 months (i.e. directly following the final smoking cessation training session), t=3 months, t=6 months, and t=12 months, respectively. Hence, the maximum total amount of incentives that can be earned is €350.
2. The descending scheme. Offers vouchers with a monetary value of €150, €100, and €50 upon validated abstinence at t=0 months, t=3 months, and t=6 months, respectively. No further incentives are provided at t=12 months. The maximum total amount of incentives is €300.
3. The ascending scheme. Offers vouchers with a monetary value of €50 and €350 upon validated abstinence t=6 months and t=12 months, respectively. No incentives are provided at t=0 and t=3 months. The maximum total amount of incentives is €400.
4. The deposit based scheme. Participants are asked to provide a €100 deposit at the beginning of the trial. The deposit scheme offers vouchers with a monetary value of €50 upon validated abstinence at both t=0 and t=3 months. Upon validated abstinence at t=6 months, participants in this scheme will receive vouchers representing a monetary value of €150; in addition, their €100 deposit will be refunded. Upon validated abstinence, at t=12 months they will receive vouchers representing a monetary value of €200. Accordingly, the maximum total amount of incentives is €450, excluding the deposit refund. If one does not stay abstinent until six months after the last training session the deposit will be donated to the Netherlands Lung Foundation.

The key aspect of the intervention is the personalisation of the incentives. The varying values over time and varying total reward sizes allow for differences in reward preferences. For example, those who prefer to earn money sooner can choose accordingly (descending scheme), however, in that case the maximum total amount rewarded will be lower, while patience is rewarded with a higher total amount (ascending scheme). Halpern et al.[10] showed that in the context of smoking cessation promotion at the workplace, deposit schemes are unpopular but very effective; therefore the current deposit scheme is designed to be the most attractive by having the largest potential total reward.

In an attempt to maximise the effectiveness of incentive provision, participants receive automated advice regarding which scheme is most likely to fit their personal characteristics. This advice will be based on the following characteristics:

For peer review only

For peer review only - http://bmjopen.bmj.com/site/about/guidelines.xhtml

1. Degree of tobacco dependence. Based on the Fagerström Test for Nicotine Dependence.[17] Fagerström score below 5 = No/low nicotine dependence, or Fagerström score of 5 or higher = nicotine dependent;
2. Readiness-to-quit. Based on Prochaska Stage of change.[18] Preparation stage (readiness to quit within the next 30 days), or (pre-)contemplation stage (ready to quit within the next six months or not ready to quit at all);[10,18]
3. Present bias. Measured by temporal discounting magnitude based on the Kirby scale.[19] Temporal discounting refers to the phenomenon where future rewards are perceived as being worth less than an immediate reward.[19] Based on a 27-item monetary choice questionnaire, those choosing more than 50% of the times the larger delayed rewards are classified as having delayed reward preference and those choosing less than 50% of the times the larger delayed rewards are classified as having present reward preference;[19,20]

and

4. willingness to pay a deposit, yes or no

The first three factors have been associated with the effectiveness of smoking cessation interventions and with reaching sustained abstinence in reward-based programs.[14] The fourth factor is used as an indicator of acceptability of the deposit-based scheme, as deposit schemes are expected to be the most effective but also the least popular. The four factors are dichotomised into "positive" or "negative" values in order to advise participants. Positive values are associated with higher quit percentages, i.e. being in a preparation stage, being low tobacco dependent and having a delayed reward preference. Negative values are associated with lower quit percentages, i.e. being in a (pre-)contemplation stage, being dependent on tobacco and having an immediate reward preference.

A combination of three positive values leads to the ascending scheme, two positive values to the standard scheme and two or three negative values to the descending scheme (see table 1). Those willing to pay a deposit are automatically advised the deposit-based scheme. The rationale behind this is that those with the lowest scores are likely to experience the most trouble quitting, and hence arguably benefit most from higher rewards in the beginning. Those with a higher score might rather benefit from incentives as a motivator to remain abstinent, and thus receive higher rewards towards the end of the study period. Importantly, participants are however free to choose the scheme of their preference when entering the trial regardless of the advice they received.

Table 1: Answer combinations and advised schemes

Degree of tobacco dependence*	Readiness-to-quit**	Present bias***	Willing to pay a deposit	Scheme advice
< 5	Ready to quit within the next 30 days	Delayed reward preference	No	Ascending
< 5	Ready to quit within the next 30 days	Present reward preference	No	Standard

For peer review only - http://bmjopen.bmj.com/site/about/guidelines.xhtml

< 5	Ready to quit in the next 6 months	Delayed reward preference	No	Standard
< 5	Ready to quit in the next 6 months	Present reward preference	No	Descending
≥ 5	Ready to quit within the next 30 days	Delayed reward preference	No	Standard
≥ 5	Ready to quit within the next 30 days	Present reward preference	No	Descending
≥ 5	Ready to quit in the next 6 months	Delayed reward preference	No	Descending
≥ 5	Ready to quit in the next 6 months	Present reward preference	No	Descending
Any answer	Any answer	Any answer	Yes	Deposit

** assessed via Fagerström score*

*** assessed via Prochaska state of change*

****assessed via Kirby discontinuity score*

As participants have freedom of choice over the incentive schemes, this is likely to result in a skewed distribution of participants over the schemes. However, this is not an issue in the current trial as the aim is to estimate the effect of personalised incentives compared to no incentives and not to estimate the discrete effects of the individual schemes. In fact, the current trial will provide information as to which schemes are most popular, how this relates to personal characteristics, and whether participants may be nudged into the scheme that is assumed to best fit their personal characteristics.

Outcomes

The primary outcome is CO-validated twelve month continuous abstinence from smoking (Russel's Standard, RS12).[21] This is assessed via self-reported abstinence and biochemically validated using expired air (CO) measurements. Self-reported abstinence assesses both point- and continuous abstinence. Point prevalence is assessed by seven-day abstinence at each follow-up point and prolonged abstinence via the total continuous abstinence period.[21] Self-reported abstinence is validated via assessment of expired air CO concentrations using a handheld monitor (PiCO™ Smokerlyzer, Bedfont Scientific Ltd., Kent, England). An exhaled-air CO reading of <10 ppm is considered indicative of abstinence.[21] CO quantification is a reliable and non-invasive method to ascertain recent smoking and the preferred method in populations using nicotine replacement

For peer review only - http://bmjopen.bmj.com/site/about/guidelines.xhtml

therapy.[21] In case of disagreement between self-reported abstinence and CO reading, abstinence is defined by the latter.

Secondary outcomes are smoking abstinence directly after the group-based smoking cessation training (t=0), and after three months (t=3), after six months (t=6) and after fifteen months (t=15). The same methods and definitions to determine continuous abstinence are used for all outcomes.

Sample size

The CATCH trial, which employed similar paying schemes as the standard arm of the current trial, reported 41.1% abstinence at twelve months follow up in the intervention arm, versus 26.4% among participants who only attended the training sessions.[13] The PERSIST trial will be embedded in health care institutions, many of which are currently implementing smoke-free policies, which is expected to be an extra external motivator for cessation. As such the validated abstinence percentages are expected to be slightly higher than in the CATCH trial, i.e. 45% and 30%, respectively. We expect that personalisation of the incentives will further increase effectiveness to approximately 50%. In order to detect this difference in validated sustained abstinence (i.e. 50% vs. 30%) with a power of 0.8 and an alpha of 0.05 and using 1:1 randomisation, a total of at least 186 participants is needed. Taking into account a 15% loss to follow-up due to unexpected employee turnover,[11,22] 220 participants are required. Further taking into account the potential maximum deviation from a 1:1 allocation ratio which may arise due to the stratified block randomisation procedure used, the minimum sample size required will increase in proportion to the number of different hospitals included in the trial. As the trial aims to estimate the effect of personalised incentives compared to no incentives and not to estimate the effect of each individual scheme, skewed allocation among the schemes is not an issue and is therefore not accounted for in the required sample size.

Recruitment

Recruitment takes place at participating health care institutions, starting with Erasmus MC. Potential participants are informed about the group-based smoking cessation training sessions through internal websites, flyers, information sessions provided by SineFuma or by referral through their company doctor. Potential participants receive a letter explaining the different incentive schemes and the research procedures. Upon enrolment, informed consent is required.

Allocation

Within participating institutions, participants will be individually randomised 1:1 to either the control or intervention arm. Permuted block randomisation with varying block sizes of four and six is used. There are two stratification factors: participating institution and readiness-to-quit (preparation or contemplation stage). A computer-generated allocation sequence was provided by ALEA randomisation service, in collaboration with the Erasmus MC Clinical Trial Centre. The block sizes will not be disclosed so as to ensure allocation concealment. ALEA will not release the randomisation result until the patient has been recruited into the trial and all baseline questionnaires have been completed. Participants are informed about their allocation by email. Inherent to the intervention and study design, participants and outcome assessors will not be blinded to allocation. Group session trainers will be blinded to allocation and data will be anonymised before analysis.

Collection methods

As described in the section 'Outcomes', self-reported abstinence will be validated using expired air CO concentration readings obtained via a handheld monitor. Participants will be invited to complete web-

For peer review only - http://bmjopen.bmj.com/site/about/guidelines.xhtml

based questionnaires assessing self-reported quality of life (EQ-5DF),[23] smoking abstinence self-efficacy (SASEQ),[24] perceived stress (Perceived Stress Scale)[25] and self-reported smoking abstinence (point prevalence and continuous abstinence)[21] at each time point during follow-up. At baseline the following items will be collected: educational level following the International Standard Classification of Education (ISCED),[26] 0 (early childhood education) to 8 (doctoral or equivalent level), individual gross monthly income (<€1500, €1500-1900, €1900-2600, >€2600), age, Body Mass Index (BMI), gender and smoking behaviour (pack years, history of quit attempts, and smoking history). In order to provide a personalised advise the following questionnaires are also taken at baseline: nicotine dependency (Fagerström scale),[17] readiness-to-quit (Prochaska stages of change)[18] and present bias (Kirby discontinuity scale).[19,27] See table 2 for a schematic overview of data collection during the trial.

Participants will be sent reminders after five days for uncompleted questionnaires. A follow-up reminder will be sent two days later. If there are still open items, participants will be contacted by telephone two days after the last reminder and a follow-up phone call will be made another two days later.

Table 2: Schematic overview of data collection during the trial.

Questionnaire	Baseline	t = 0 months	t = 3 months	t = 6 months	t = 12 months *	t = 15 months
Demographic variables	X					
Fagerström scale	X					
Prochaska stage of change	X					
Kirby discontinuity scale	X					
Willingness to pay a deposit	X					
EQ-5DF**	X	X	X	X	X	X
SASEQ***	X	X	X	X	X	X
Perceived stress scale	X	X	X	X	X	X
Self-reported abstinence*** *	X	X	X	X	X	X
Evaluation training sessions		X				
Evaluation PERSIST						X

**t = 12 months is the primary endpoint*

*** EurQol Five Dimensions Health Questionnaire*

**** Smoking abstinence self-efficacy questionnaire*

***** The self-reported abstinence questionnaire is adjusted to each corresponding time moment, e.g. at t =3 the maximum period not smoked is three months etc.*

Data management

Informed consent will be provided digitally through LimeSurvey (LimeSurvey GmbH, Hamburg, Germany). All trial data will be entered electronically using GemsTracker (GemsTracker ©, Erasmus MC and *Equipe Zorgbedrijven*, Rotterdam, the Netherlands). Data are handled confidentially and stored anonymously. Where data need to be linked to an individual subject (e.g. linking longitudinal data), a subject identification code will be used. The code will not be based on patient initials and date of birth to preserve anonymity. The key to the code will be safeguarded by the investigator and kept separate from data files. All of the study essential documents will be retained and archived for 15 years after completion of the study. They will be stored securely and adequately protected from damage.

Statistical methods

The primary analysis compares continuous smoking abstinence rates at twelve months after the group-based training session in the intervention arm against the control arm, following an intention-to-treat analysis. Secondary continuous smoking abstinence at fifteen months is assessed to see if abstinence is sustained after the incentives stopped. All randomised participants will be included in the final analysis, those lost to follow up will be classified as smoking except for an unavoidable loss to follow up (e.g. untraceable contact information or death, following the Russel standard).[21] Generalised linear models with a logit link function will be used to assess the difference in the primary outcome between control and intervention arm at t =12 months and to account for clustering between hospitals.

Additionally, subgroup analyses will be performed to identify possible subgroup-specific effects of the intervention. Planned subgroup analyses are based on:

1. readiness-to-quit. Preparation stage versus contemplation stage;[10,18]
2. degree of tobacco dependence. Fagerström score below 5 versus Fagerström score of 5 or higher;[17]
3. present bias. Delayed versus present reward preference. Delayed reward preference = more than 50% larger delayed reward is chosen, or present reward preference = less than 50% larger delayed reward is chosen; [19,20]
4. income. Based on gross monthly income in €. Low income = below 1500 versus moderate/high income = above 1500.[28]

and

5. Educational level. Based on the International Classification of Education (ISCED).[26] Lower educated = ISCED 1-4 versus higher educated = ISCED 5-8.

Subgroup analysis are not included in the power analysis and therefore expected to be suggestive. For missing data on subgroup information, multiple imputation will be performed.

For peer review only - http://bmjopen.bmj.com/site/about/guidelines.xhtml

Patient and Public Involvement

Participants were not involved in designing the trial. Once the results are published participants will be informed about the publication. This email will be drafted suitable for a non-specialist audience. Public involvement will be sought through the Erasmus Initiative Smarter Choices for Better Health.

ETHICS AND DISSEMINATION

The study protocol has been assessed by the Erasmus MC Medical Ethics Committee (MEC-2019-0140). The committee decided that according to the Dutch Human Research Law (WMO) the protocol required no formal ethical approval. All participants will provide informed consent for participating in the study.

The results will be published in a peer-reviewed scientific journal. On completion of the trial, and after the publication of the results, data requests can be submitted to the corresponding author at Erasmus MC, Rotterdam, the Netherlands.

DISCUSSION

We present the protocol of the "PERSonalised Incentives for Supporting Tobacco cessation" (PERSIST) trial. The aim of the trial is to evaluate the effectiveness of personalised incentives in addition to group-based smoking cessation training on sustained smoking abstinence among hospital employees compared to group-based training alone. Personalisation entails that participants are advised and can choose from four different incentive schemes: standard, ascending, descending, and deposit-based.

Strengths and limitations

The PERSIST trial is unique in its approach to personalising incentives to promote smoking cessation. Strong links between personal characteristics and effectiveness of incentives to promote smoking cessation have been demonstrated previously, however, to our knowledge, the effectiveness of personalised incentives has not been studied before.[14] Another unique element of the PERSIST trial is that participants receive personalised advice for an incentive scheme, based on personal characteristics, yet are free to deviate from the advised scheme. Voluntary choice with a personalised default is a common tool in behavioural economics to nudge people towards the scheme that is plausibly in their best interest.[29] This feature is scientifically innovative in the context of smoking cessation programmes, and importantly let the treated individuals to choose the scheme they will be enrolled.

By focusing on supporting smoking cessation among hospital employees, the PERSIST trial is executed in a highly relevant setting as in light of the National Prevention Agreement all university hospitals have to be smoke free by 2020 and all general hospitals by 2025.[30] Finally, the PERSIST trial progresses from previous studies by extending follow-up beyond the last time point when incentives are provided to investigate if participants remain abstinent when incentives are no longer provided.

A limitation of the PERSIST trial is that participants from the intervention and control arm attend the same cessation support training sessions due to practical reasons, as slow inclusion rates otherwise would have caused long waiting periods between signing up and starting the training. Participants in the control arm might perceive this negatively and potentially drop out, causing attrition bias. Another consequence could be spill-over effects;[31] sharing of vouchers between control and intervention arm, leading to a double underestimation of treatment effectiveness. To avoid this, participants are informed about their status as early as possible to make sure they have time to accept their status. A third arm consisting of non-personalised incentives could have provided additional potentially

For peer review only - http://bmjopen.bmj.com/site/about/guidelines.xhtml

valuable information on the effectiveness of personalised incentives versus incentives provided according to a fixed scheme. However, this option would have required unfeasibly large sample size.

CONCLUSION

The PERSIST trial is the first in its kind to personalise incentives to support sustained smoking cessation and unique in its focus on hospital employees. Results of the trial can provide insight into the effective elements of incentives for behavioural change.

Availability of data and material

Data will become available upon completion of the study

Declarations

- Consent for publication
  Not applicable, no personal or identifying data will be published.
- Access to the data
  Data will become available upon completion of the study, which is expected to be beginning of 2023.
- Amendments
  Important protocol modifications will be communicated to the METC and trial participants
- Competing interests
  The authors declare that they have no competing interests
- Funding
  This study is funded by the Erasmus Initiative Smarter Choices for Better Health and the Erasmus Trustfonds. JVB is funded by a personal fellowship from the Netherlands Lung Foundation.
- Author Contributions
  JVB, FvL and HvK wrote the initial version of the programme grant request on which this protocol was based. NWB drafted the manuscript and runs the day-to-day study. DTOC did the sample size calculations, advised on the randomisation method and the allocation algorithm and MKR advised on the statistical analysis. The paper was revised and edited by all authors and all authors approved the final manuscript.

REFERENCES

1. Forouzanfar MH, Afshin A, Alexander LT, et al. Global, regional, and national comparative risk assessment of 79 behavioural, environmental and occupational, and metabolic risks or clusters of risks, 1990–2015: a systematic analysis for the Global Burden of Disease Study 2015. *Lancet* 2016;**388**(10053):1659-724.
2. Nayak P, Pechacek TF, Slovic P, et al. Regretting Ever Starting to Smoke: Results from a 2014 National Survey. *Int J Environ Res Public Health* 2017;**14**(4):12.
3. Netherlands S. Leefstijl en (preventief) gezondheidsonderzoek; persoonskenmerken. 19-03-2019 ed: CBS, 2019.
4. Stead LF, Perera R, Bullen C, et al. Nicotine replacement therapy for smoking cessation. *Cochrane Database Syst Rev* 2012(11).
5. Lancaster T, Stead LF. Individual behavioural counselling for smoking cessation. *Cochrane Database Syst Rev* 2017(3).

For peer review only - http://bmjopen.bmj.com/site/about/guidelines.xhtml

6. Stead LF, Carroll AJ, Lancaster T. Group behaviour therapy programmes for smoking cessation. *Cochrane Database Syst Rev* 2017(3).
7. Notley C, Gentry S, Livingstone-Banks J, et al. Incentives for smoking cessation. *Cochrane Database Syst Rev* 2019(7).
8. Odum AL. Delay discounting: I'm a k, you're a k. *J Exp Anal Behav* 2011;**96**(3):427-39.
9. O'Donoghue T, Rabin M. Doing it now or later. *Am Econ Rev* 1999;**89**(1):103-24.
10. Halpern SD, French B, Small DS, et al. Randomized trial of four financial-incentive programs for smoking cessation. *N Engl J Med* 2015;**372**(22):2108-17.
11. Volpp KG, Troxel AB, Pauly MV, et al. A randomized, controlled trial of financial incentives for smoking cessation. *N Engl J Med* 2009;**360**(7):699-709.
12. Halpern SD, Harhay MO, Saulsgiver K, et al. A Pragmatic Trial of E-Cigarettes, Incentives, and Drugs for Smoking Cessation. *N Engl J Med* 2018;**378**(24):2302-10.
13. van den Brand FA, Nagelhout GE, Winkens B, et al. Effect of a workplace-based group training programme combined with financial incentives on smoking cessation: a cluster-randomised controlled trial. *Lancet Public Health* 2018;**3**(11):e536-e44.
14. Halpern SD, French B, Small DS, et al. Heterogeneity in the Effects of Reward- and Deposit-based Financial Incentives on Smoking Cessation. *Am J Respir Crit Care Med* 2016;**194**(8):981-88.
15. Chan A-W, Tetzlaff JM, Altman DG, et al. SPIRIT 2013: new guidance for content of clinical trial protocols. *Lancet* 2013;**381**(9861):91-92.
16. Milieu RvVe. Loket gezond leven *RIVM* 2019 [Available from: https://interventies.loketgezondleven.nl/leefstijlinterventies/interventies-zoeken/1400743.
17. Heatherton TF, Kozlowski LT, Frecker RC, et al. The Fagerstrom Test for Nicotine Dependence: a revision of the Fagerstrom Tolerance Questionnaire. *Br J Addict* 1991;**86**(9):1119-27.
18. Prochaska JO, Velicer WF, DiClemente CC, et al. Measuring processes of change: applications to the cessation of smoking. *J Consult Clin Psychol* 1988;**56**(4):520-8.
19. Kirby KN. Bidding on the future: Evidence against normative discounting of delayed rewards. *J Exp Psychol Gen* 1997;**126**(1):54-70.
20. Kaplan BA, Amlung M, Reed DD, et al. Automating Scoring of Delay Discounting for the 21- and 27-Item Monetary Choice Questionnaires. *Behav Analyst* 2016;**293-304**(2):12.
21. West R, Hajek P, Stead L, et al. Outcome criteria in smoking cessation trials: proposal for a common standard. *Addiction* 2005;**100**(3):299-303.
22. van den Brand FA, Nagelhout GE, Winkens B, et al. Effect of a workplace-based group training programme combined with financial incentives on smoking cessation: a cluster-randomised controlled trial. *Lancet Public Health* 2018;**3**(11):9.
23. Herdman M, Gudex C, Lloyd A, et al. Development and preliminary testing of the new five-level version of EQ-5D (EQ-5D-5L). *Qual Life Res* 2011;**20**(10):1727-36.
24. Spek V, Lemmens F, Chatrou M, et al. Development of a smoking abstinence self-efficacy questionnaire. *Int J Behav Med* 2013;**20**(3):444-9.
25. Cohen S, Kamarck T, Mermelstein R. A global measure of perceived stress. *J Health Soc Behav* 1983;**24**(4):385-96.
26. Unesco Institute for Statistics. International standard classification of education: ISCED 2011: UNESCO Institute for Statistics Montreal, 2012.
27. Gray JC, Amlung MT, Palmer AA, et al. Syntax for calculation of discounting indices from the monetary choice questionnaire and probability discounting questionnaire. *J Exp Anal Behav* 2016;**106**(2):156-63.
28. Netherlands S. Inkomensgroepen: Particuliere huishoudens: *Statistics Netherlands*; 2014 [updated 15-04-2018. Available from: https://statline.cbs.nl/Statweb/publication/?DM=SLNL&PA=71013ned&D1=0-2&D2=1-2&D3=11-14&D4=44-62&D5=l&HDR=G4,G1,T&STB=G3,G2&VW=L.
29. Schlag P. Nudge, choice architecture, and libertarian paternalism: HeinOnline, 2009.
30. The National Prevention Agreement In: Ministry of Health WaS, ed. The Hague 2019.

For peer review only - http://bmjopen.bmj.com/site/about/guidelines.xhtml

31. Angelucci M, Maro VD. Program evaluation and spillover effects: The World Bank, 2015.

For peer review only

For peer review only - http://bmjopen.bmj.com/site/about/guidelines.xhtml

For peer review only

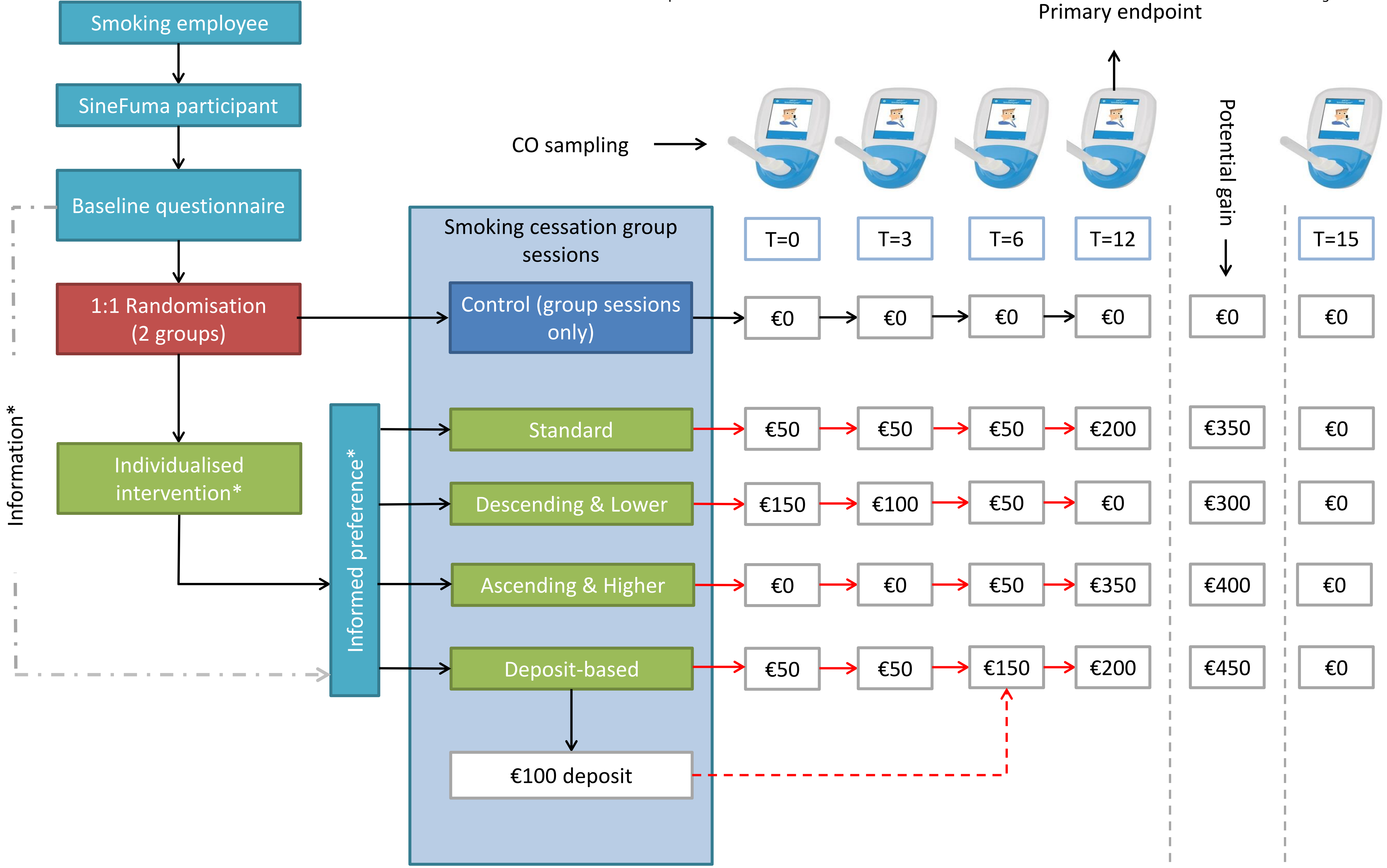

*Participants are provided with an informed choice regarding the individualised incentive scheme based on: 1. degree of tobacco dependence, 2. Readiness to quit and 3. Present-bias.
Note: red arrows are conditional on sustained biochemically validated smoking cessation

PERSIST – SPIRIT CHECKLIST

Section/item	ItemNo	Description	Page number
**Administrative information**			
Title	1	Descriptive title identifying the study design, population, interventions, and, if applicable, trial acronym	1
Trial registration	2a	Trial identifier and registry name. If not yet registered, name of intended registry	2
	2b	All items from the World Health Organization Trial Registration Data Set	NA
Protocol version	3	Date and version identifier	
Funding	4	Sources and types of financial, material, and other support	14
Roles and responsibilities	5a	Names, affiliations, and roles of protocol contributors	1
	5b	Name and contact information for the trial sponsor	NA
	5c	Role of study sponsor and funders, if any, in study design; collection, management, analysis, and interpretation of data; writing of the report; and the decision to submit the report for publication, including whether they will have ultimate authority over any of these activities	NA
	5d	Composition, roles, and responsibilities of the coordinating centre, steering committee, endpoint adjudication committee, data management team, and other individuals or groups overseeing the trial, if applicable (see Item 21a for data monitoring committee)	NA
**Introduction**			
Background and rationale	6a	Description of research question and justification for undertaking the trial, including summary of relevant studies (published and unpublished) examining benefits and harms for each intervention	4
	6b	Explanation for choice of comparators	NA
Objectives	7	Specific objectives or hypotheses	5

For peer review only - http://bmjopen.bmj.com/site/about/guidelines.xhtml

Trial design	8	Description of trial design including type of trial (eg, parallel group, crossover, factorial, single group), allocation ratio, and framework (eg, superiority, equivalence, noninferiority, exploratory)	5
**Methods: Participants, interventions, and outcomes**			
Study setting	9	Description of study settings (eg, community clinic, academic hospital) and list of countries where data will be collected. Reference to where list of study sites can be obtained	5
Eligibility criteria	10	Inclusion and exclusion criteria for participants. If applicable, eligibility criteria for study centres and individuals who will perform the interventions (eg, surgeons, psychotherapists)	5
Interventions	11a	Interventions for each group with sufficient detail to allow replication, including how and when they will be administered	5
	11b	Criteria for discontinuing or modifying allocated interventions for a given trial participant (eg, drug dose change in response to harms, participant request, or improving/worsening disease)	NA
	11c	Strategies to improve adherence to intervention protocols, and any procedures for monitoring adherence (eg, drug tablet return, laboratory tests)	NA
	11d	Relevant concomitant care and interventions that are permitted or prohibited during the trial	NA
Outcomes	12	Primary, secondary, and other outcomes, including the specific measurement variable (eg, systolic blood pressure), analysis metric (eg, change from baseline, final value, time to event), method of aggregation (eg, median, proportion), and time point for each outcome. Explanation of the clinical relevance of chosen efficacy and harm outcomes is strongly recommended	9
Participant timeline	13	Time schedule of enrolment, interventions (including any run-ins and washouts), assessments, and visits for participants. A schematic diagram is highly recommended (see Figure)	6

For peer review only - http://bmjopen.bmj.com/site/about/guidelines.xhtml

Sample size	14	Estimated number of participants needed to achieve study objectives and how it was determined, including clinical and statistical assumptions supporting any sample size calculations	9
Recruitment	15	Strategies for achieving adequate participant enrolment to reach target sample size	10
**Methods: Assignment of interventions (for controlled trials)**			
Allocation:			10
Sequence generation	16a	Method of generating the allocation sequence (eg, computer-generated random numbers), and list of any factors for stratification. To reduce predictability of a random sequence, details of any planned restriction (eg, blocking) should be provided in a separate document that is unavailable to those who enrol participants or assign interventions	10
Allocation concealment mechanism	16b	Mechanism of implementing the allocation sequence (eg, central telephone; sequentially numbered, opaque, sealed envelopes), describing any steps to conceal the sequence until interventions are assigned	10
Implementation	16c	Who will generate the allocation sequence, who will enrol participants, and who will assign participants to interventions	10
Blinding (masking)	17a	Who will be blinded after assignment to interventions (eg, trial participants, care providers, outcome assessors, data analysts), and how	10
	17b	If blinded, circumstances under which unblinding is permissible, and procedure for revealing a participant's allocated intervention during the trial	NA
**Methods: Data collection, management, and analysis**			

For peer review only - http://bmjopen.bmj.com/site/about/guidelines.xhtml

Data collection methods	18a	Plans for assessment and collection of outcome, baseline, and other trial data, including any related processes to promote data quality (eg, duplicate measurements, training of assessors) and a description of study instruments (eg, questionnaires, laboratory tests) along with their reliability and validity, if known. Reference to where data collection forms can be found, if not in the protocol	10
	18b	Plans to promote participant retention and complete follow-up, including list of any outcome data to be collected for participants who discontinue or deviate from intervention protocols	10
Data management	19	Plans for data entry, coding, security, and storage, including any related processes to promote data quality (eg, double data entry; range checks for data values). Reference to where details of data management procedures can be found, if not in the protocol	11
Statistical methods	20a	Statistical methods for analysing primary and secondary outcomes. Reference to where other details of the statistical analysis plan can be found, if not in the protocol	12
	20b	Methods for any additional analyses (eg, subgroup and adjusted analyses)	12
	20c	Definition of analysis population relating to protocol non-adherence (eg, as randomised analysis), and any statistical methods to handle missing data (eg, multiple imputation)	12
**Methods: Monitoring**			
Data monitoring	21a	Composition of data monitoring committee (DMC); summary of its role and reporting structure; statement of whether it is independent from the sponsor and competing interests; and reference to where further details about its charter can be found, if not in the protocol. Alternatively, an explanation of why a DMC is not needed	12
	21b	Description of any interim analyses and stopping guidelines, including who will have access to these interim results and make the final decision to terminate the trial	NA

Harms	22	Plans for collecting, assessing, reporting, and managing solicited and spontaneously reported adverse events and other unintended effects of trial interventions or trial conduct	NA
Auditing	23	Frequency and procedures for auditing trial conduct, if any, and whether the process will be independent from investigators and the sponsor	NA
**Ethics and dissemination**			
Research ethics approval	24	Plans for seeking research ethics committee/institutional review board (REC/IRB) approval	12
Protocol amendments	25	Plans for communicating important protocol modifications (eg, changes to eligibility criteria, outcomes, analyses) to relevant parties (eg, investigators, REC/IRBs, trial participants, trial registries, journals, regulators)	14
Consent or assent	26a	Who will obtain informed consent or assent from potential trial participants or authorised surrogates, and how (see Item 32)	11
	26b	Additional consent provisions for collection and use of participant data and biological specimens in ancillary studies, if applicable	NA
Confidentiality	27	How personal information about potential and enrolled participants will be collected, shared, and maintained in order to protect confidentiality before, during, and after the trial	11
Declaration of interests	28	Financial and other competing interests for principal investigators for the overall trial and each study site	14
Access to data	29	Statement of who will have access to the final trial dataset, and disclosure of contractual agreements that limit such access for investigators	14
Ancillary and post-trial care	30	Provisions, if any, for ancillary and post-trial care, and for compensation to those who suffer harm from trial participation	NA

For peer review only

Dissemination policy	31a	Plans for investigators and sponsor to communicate trial results to participants, healthcare professionals, the public, and other relevant groups (eg, via publication, reporting in results databases, or other data sharing arrangements), including any publication restrictions	12
	31b	Authorship eligibility guidelines and any intended use of professional writers	14
	31c	Plans, if any, for granting public access to the full protocol, participant-level dataset, and statistical code	NA
**Appendices**			
Informed consent materials	32	Model consent form and other related documentation given to participants and authorised surrogates	17
Biological specimens	33	Plans for collection, laboratory evaluation, and storage of biological specimens for genetic or molecular analysis in the current trial and for future use in ancillary studies, if applicable	NA

For peer review only

For peer review only - http://bmjopen.bmj.com/site/about/guidelines.xhtml

BMJ Open

Personalised incentives for supporting tobacco cessation among health care employees (PERSIST): a randomised controlled trial protocol

Journal:	*BMJ Open*
Manuscript ID	bmjopen-2020-037799.R1
Article Type:	Protocol
Date Submitted by the Author:	18-May-2020
Complete List of Authors:	Boderie, Nienke; Erasmus University Medical Center, Department of Public Health<br>van Kippersluis, Hans; Erasmus University Rotterdam Erasmus School of Economics; Tinbergen Institute<br>Ó Ceallaigh, Diarmaid; Erasmus University Rotterdam Erasmus School of Economics<br>Rado, Márta; Erasmus University Medical Centre, Department of Public Health; Erasmus University Medical Centre–Sophia Children's Hospital, Paediatrics<br>Burdorf, Alex; Erasmus University Medical Centre, Department of Public Health<br>Van Lenthe, Frank; Erasmus University Medical Centre, Department of Public Health<br>Been, JV; Erasmus University Medical Centre–Sophia Children's Hospital, Paediatrics, division of Neonatology; Erasmus University Medical Centre, Department of Public Health
**Primary Subject Heading**:	Smoking and tobacco
Secondary Subject Heading:	Public health
Keywords:	PUBLIC HEALTH, SOCIAL MEDICINE, HEALTH ECONOMICS

SCHOLARONE™ Manuscripts

I, the Submitting Author has the right to grant and does grant on behalf of all authors of the Work (as defined in the below author licence), an exclusive licence and/or a non-exclusive licence for contributions from authors who are: i) UK Crown employees; ii) where BMJ has agreed a CC-BY licence shall apply, and/or iii) in accordance with the terms applicable for US Federal Government officers or employees acting as part of their official duties; on a worldwide, perpetual, irrevocable, royalty-free basis to BMJ Publishing Group Ltd ("BMJ") its licensees and where the relevant Journal is co-owned by BMJ to the co-owners of the Journal, to publish the Work in this journal and any other BMJ products and to exploit all rights, as set out in our licence.

The Submitting Author accepts and understands that any supply made under these terms is made by BMJ to the Submitting Author unless you are acting as an employee on behalf of your employer or a postgraduate student of an affiliated institution which is paying any applicable article publishing charge ("APC") for Open Access articles. Where the Submitting Author wishes to make the Work available on an Open Access basis (and intends to pay the relevant APC), the terms of reuse of such Open Access shall be governed by a Creative Commons licence – details of these licences and which Creative Commons licence will apply to this Work are set out in our licence referred to above.

Other than as permitted in any relevant BMJ Author's Self Archiving Policies, I confirm this Work has not been accepted for publication elsewhere, is not being considered for publication elsewhere and does not duplicate material already published. I confirm all authors consent to publication of this Work and authorise the granting of this licence.

For peer review only

BMJ Open

Personalised incentives for supporting tobacco cessation among health care employees (PERSIST): a randomised controlled trial protocol

Nienke W Boderie[1], Hans van Kippersluis[2,3], Diarmaid T Ó Ceallaigh[2], Márta K. Radó[1,4], Alex Burdorf[1], Frank J van Lenthe[1], Jasper V Been[1,4]

[1] Department of Public Health, Erasmus MC, University Medical Centre Rotterdam, Rotterdam, The Netherlands

[2] Erasmus School of Economics, Erasmus University Rotterdam, Rotterdam, The Netherlands

[3] Tinbergen Institute, Rotterdam, The Netherlands

[4] Department of Paediatrics, division of Neonatology, Erasmus MC - Sophia Children's Hospital, University Medical Centre Rotterdam, Rotterdam, The Netherlands

Corresponding author:

Dr. Jasper Been

Doctor Molewaterplein 40, 3015 GD Rotterdam

j.been@erasmusmc.nl

+ 31 10 36077

Word count: 4502

For peer review only - http://bmjopen.bmj.com/site/about/guidelines.xhtml

For peer review only

KEY WORDS

Group based training - Financial Incentives - Personalised - Smoking cessation - Validated

ABSTRACT (312 words)

Background: Smoking is the primary preventable risk factor for disease and premature mortality. It is highly addictive and cessation attempts are often unsuccessful. Incentive-based programmes may be an effective method to reach sustained abstinence. Individualisation of incentives based on personal characteristics yields potential to further increase effectiveness of incentive-based programmes.

Method: A randomised controlled trial among health care workers, recruited through their employer and signed up for a group-based smoking cessation programme. The intervention under study is the provision of personalised incentives upon validated smoking cessation at several time points after the smoking-cessation programme. A total of 220 participants is required. Participants are randomised 1:1 into intervention (personalised incentives) or control (no incentives). All participants join the group-based programme. Incentives are provided upon validated abstinence directly after the smoking cessation programme and after three, six and twelve months.

Incentives are provided according to four schemes: (1) Standard: total reward size €350, pay-out scheme: €50, €50, €50 and €200, (2) Descending: total reward size €300, pay-out scheme: €150, €100, €50 and €0, (3) Ascending: total reward size: €400, pay-out scheme: €0, €0, €50, €350, and (4) Deposit: total reward size €450, pay-out scheme: €50, €50, €150, €200; participants pay a €100 deposit, returned conditional on abstinence after six months.

Advice on which incentive scheme suits participants best is based on readiness to quit, nicotine dependency and long or short-term reward preference. Participants are free to deviate from this advice. Abstinence is validated at each time point, with 15-month total follow-up. The primary end-point is validated abstinence at twelve months. Effectiveness will be determined by intention-to-treat analysis.

Ethics and dissemination: The Erasmus MC Medical Ethics Committee decided that according to the Dutch Human Research Law (WMO) the protocol required no formal ethical approval. The results will be published in a peer-reviewed scientific journal and communicated to the participants.

Trial registration: Netherlands Trial Register NL7711

For peer review only

STRENGTHS AND LIMITATIONS

- The PERSIST trial has a unique hybrid design with a nudged choice embedded within a randomised trial, and is the first of its kind to provide a personalised advise on the mode of providing incentives which likely best fits individual participant characteristics.
- This trial furthermore addresses a knowledge gap regarding long-term effectiveness of incentive-based strategies beyond the actual intervention period
- A focus on helping hospital employees quit smoking is essential given their exemplary role in promoting health.
- For logistical reasons participants from the intervention and control arm may attend the same group sessions, potentially leading to spill-over effects.
- Due to the predicted sample size, it is not possible to include an additional intervention arm where incentives are provided but not personalised

For peer review only - http://bmjopen.bmj.com/site/about/guidelines.xhtml

For peer review only

For peer review only - http://bmjopen.bmj.com/site/about/guidelines.xhtml

BACKGROUND

Smoking is the primary preventable risk factor for disease and premature mortality.[1] Smoking is highly addictive and the vast majority of cessation attempts are unsuccessful.[2] In 2017, 38% of smokers in the Netherlands attempted to quit for at least 24 hours.[3] Several methods exist to increase the odds of quitting, such as nicotine replacement therapy,[4] individual behavioural counselling,[5] and group-based smoking cessation programmes.[6] However, despite the relative effectiveness of these studies, in absolute numbers the proportion of smokers that quit is still low.

A promising method to encourage smoking cessation is the provision of financial incentives upon validated abstinence. A recent Cochrane review found high-certainty evidence for increased smoking cessation rates at six or twelve months follow up (RR = 1.49, 95% confidence interval (CI) 1.28 – 1.73).[7] Financial incentives make the desired behaviour - in this case smoking cessation - more attractive by rewarding it. Importantly, financial incentives provide a short term reward for a behavioural change that would normally only provide rewards in the long term. Long term rewards are often valued less compared to short term rewards due to declining perceived values of a delayed reward, so-called delay discounting.[8] Typically, people give disproportionally stronger relative weights to instantaneous rewards, so-called present bias,[9] which gives a strong theoretical argument for providing short-term incentives for smoking cessation.

Incentive-based smoking cessation programmes have previously been implemented at the workplace.[10-12] Recently, the “Continuous Abstinence Through Corporate Healthcare” (CATCH) trial was carried out among 31 Dutch companies, randomising companies to group-based smoking cessation training for smoking employees with or without financial incentives to promote sustained cessation. Among those eligible to receive incentives (up to €350), the proportion of abstinent participants at one year follow-up was significantly higher compared to those assigned to group-based training alone (41% vs 26%).[13]

The effectiveness of incentives in promoting sustained smoking cessation depends on how and when rewards are provided. Traditionally incentives have been tested according to a one-size-fits-all approach, using incentive size as a measure of anticipated effectiveness. A recent US trial comparing four different incentive programs, however, showed that effectiveness is variable among different types and schemes of incentives.[10] A deposit-based scheme, where participants commit their own money and return is conditional on validated abstinence, was unpopular but resulted in a significantly higher proportion of abstinent individuals compared to the reward-based incentives arm.[10] Further analysis revealed associations between personal characteristics and the acceptability and efficacy of incentive programmes.[14] Both readiness-to-quit-smoking and low tendency to discount rewards were associated with increased effectiveness of both reward and deposit based programmes. Personalisation of incentive programmes thus yields the potential to further increase the effectiveness of incentives to promote long term abstinence.

Here we present the study protocol for the “Personalised Incentives for Supporting Tobacco Cessation” (PERSIST) trial. The trial will be conducted among health care employees, for whom smoking cessation is particularly relevant as they may be expected to have an exemplary role. Moreover, in an attempt to work towards a smoke free generation by 2040, the Dutch National Prevention Agreement requires university hospitals to be smoke-free by mid-2020 and all health care facilities by 2025.

For peer review only - http://bmjopen.bmj.com/site/about/guidelines.xhtml

The aim of this study is to investigate whether personalised incentives in addition to a group-based smoking cessation programme will increase sustained smoking cessation among participants. This unique randomised controlled trial (RCT) attempts to optimise effectiveness of incentive programmes by providing personalised advice while leaving participants in full control to choose the scheme of their choice. Our approach will further provide insight into which incentive schemes participants prefer and if and how they can be nudged into the programme that best fits their personal characteristics.

METHODS AND ANALYSIS

Objectives

The main objective is to evaluate the effectiveness of personalised incentives in combination with group-based training sessions provided in the work environment on sustained smoking abstinence among health care workers compared to group-based training sessions alone.

Trial design

The PERSIST trial is a randomised controlled non-blinded trial with two parallel arms.

Methods

The protocol follows the SPIRIT guidelines for protocol papers.[15]

Study setting

The trial will be implemented at Dutch health care institutions. The trial is primarily implemented at the Erasmus University Medical Centre (Erasmus MC) and is further extended to Franciscus Gasthuis & Vlietland and Ikazia hospital. Inclusion of other health care institutions in the greater Rotterdam area is ongoing.

Trial population and eligibility criteria

Employees are eligible if they are: (1) aged 18 years or over, (2) employed at one of the participating health care institutions, and (3) a daily smoker (i.e. smoking at least one cigarette per day). Exclusion criteria are: (1) only using e-cigarettes and (2) not being able to physically attend the group-based training sessions.

Participants are recruited through their employer. Information about the training and the PERSIST trial is spread through intranet, email, flyers and screensavers. In collaboration with the organisation responsible for the training (SineFuma, see *Interventions*) an information sessions about the training and trial are organised.

Interventions

Eligible participants are individually randomised 1:1 into a control arm, receiving no incentives, and an intervention arm, receiving personalised incentives. See Figure 1 for a flowchart. Both arms participate in a group-based smoking cessation training provided by SineFuma, a company specialised in supporting smoking cessation (www.rookvrijookjij.nl). Groups consist of eight to 16 participants and the training consists of seven sessions of approximately ninety minutes duration held over a period of eight weeks. Participants in the intervention and control arms participate in the same training sessions for logistical reasons. Group-based training sessions will start as soon as at least eight people have signed up to avoid participants lose motivation due to long waiting times.

For peer review only - http://bmjopen.bmj.com/site/about/guidelines.xhtml

Group sessions are provided at locations within each of the participating health care institutions. The aim of the training is to quit smoking as a group: participants jointly quit smoking in the third session. The follow-on sessions then focus on supporting sustained cessation through the first few weeks following the quit attempt. The participants and coach discuss varous types of nicotine replacement therapy (NRT) as part of the training. No NRT is provided in the trial but participants are free to use an NRT of their choice. During each session, exhaled carbon monoxide (CO) measurements (see outcomes for more information) are used as a motivator to stay abstinent. The SineFuma group-based training is recognised as an efficient lifestyle intervention by the Dutch Government,[16] and participation costs are reimbursed by basic insurance. As such, the control arm can be considered as usual care. If a portion of the costs are at the employee's own expense through the deductible, employers have agreed to pay for the training.

Incentives

The difference between intervention and control conditions lies within the provision of incentives. Participants in the intervention arm who remain continuously abstinent following the joint quit attempt are eligible to receive incentives according to a fixed time schedule. Incentives are provided as vouchers representing monetary value. Vouchers can be spent at online web-shops or on events such as a high tea in a local café or a night at a hotel etc. Vouchers cannot be spent on tobacco products. The first incentive is provided directly after the group-based smoking cessation training, marking the moment from which participants have to remain abstinent independently.

In order to personalise incentives, four different incentive schemes are offered, which participants are free to choose from. Across the different schemes, the monetary value of the incentives varies over time, and the potential total financial reward differs. The schemes are as follows (see figure 1):

1. The standard scheme. Analogous to the CATCH trial,[13] vouchers with a monetary value of €50, €50, €50, and €200 are offered upon validated abstinence at t=0 months (i.e. directly following the final smoking cessation training session), t=3 months, t=6 months, and t=12 months, respectively. Hence, the maximum total amount of incentives that can be earned is €350.
2. The descending scheme. Offers vouchers with a monetary value of €150, €100, and €50 upon validated abstinence at t=0 months, t=3 months, and t=6 months, respectively. No further incentives are provided at t=12 months. The maximum total amount of incentives is €300.
3. The ascending scheme. Offers vouchers with a monetary value of €50 and €350 upon validated abstinence t=6 months and t=12 months, respectively. No incentives are provided at t=0 and t=3 months. The maximum total amount of incentives is €400.
4. The deposit based scheme. Participants are asked to provide a €100 deposit at the beginning of the trial. The deposit scheme offers vouchers with a monetary value of €50 upon validated abstinence at both t=0 and t=3 months. Upon validated abstinence at t=6 months, participants in this scheme will receive vouchers representing a monetary value of €150; in addition, their €100 deposit will be refunded. Upon validated abstinence, at t=12 months they will receive vouchers representing a monetary value of €200. Accordingly, the maximum total amount of incentives is €450, excluding the deposit refund. If one does not stay abstinent until six months after the last training session the deposit will be donated to the Netherlands Lung Foundation.

In choosing the monetary amounts of incentives, we followed the general principle that the incentives should be large enough to motivate smoking cessation, but not too large either to avoid crowding out

For peer review only - http://bmjopen.bmj.com/site/about/guidelines.xhtml

intrinsic motivation and to make scaling up the trial prohibitively costly.[17] The standard scheme is identical to the scheme in the CATCH trial.[18] Given time discounting due to inflation and individual impatience, it seems natural to apply some compensation in the total potential gains to the descending and ascending scheme. For example, why would someone self-select into an ascending scheme with zero pay-out in the first six months if the total potential gain is the same as the descending scheme where one can cash-in €150,- already after at baseline? Further, as deposit contracts have proven to be most effective among those who choose one,[10] we aimed to make this scheme the most attractive by providing the largest total reward size.

The key aspect of the intervention is the personalisation of the incentives. The varying values over time and varying total reward sizes allow for differences in reward preferences. For example, those who prefer to earn money sooner can choose accordingly (descending scheme), however, in that case the maximum total amount rewarded will be lower, while patience is rewarded with a higher total amount (ascending scheme). Halpern, et al.[10] showed that in the context of smoking cessation promotion at the workplace, deposit schemes are unpopular but very effective; therefore the current deposit scheme is designed to be the most attractive by having the largest potential total reward.

In an attempt to maximise the effectiveness of incentive provision, participants receive automated advice regarding which scheme is most likely to fit their personal characteristics. This advice will be based on the following characteristics:

1. Degree of tobacco dependence. Based on the Fagerström Test for Nicotine Dependence.[19] Fagerström score below 5 = no/low nicotine dependence, or Fagerström score of 5 or higher = nicotine dependent;
2. Readiness-to-quit. Based on Prochaska Stage of change.[20] Preparation stage (readiness to quit within the next 30 days), or (pre-)contemplation stage (ready to quit within the next six months or not ready to quit at all);[10,20]
3. Present bias. Measured by temporal discounting magnitude based on the Kirby scale.[21] Temporal discounting refers to the phenomenon where future rewards are perceived as being worth less than an immediate reward.[21] Based on a 27-item monetary choice questionnaire, those choosing more than 50% of the times the larger delayed rewards are classified as having delayed reward preference and those choosing less than 50% of the times the larger delayed rewards are classified as having present reward preference;[21,22]

and

4. willingness to pay a deposit, yes or no

The first three factors have been associated with the effectiveness of smoking cessation interventions and with reaching sustained abstinence in reward-based programs.[14] The fourth factor is used as an indicator of acceptability of the deposit-based scheme, as deposit schemes are expected to be the most effective but also the least popular. The four factors are dichotomised into "positive" or "negative" values in order to advise participants. Positive values are associated with higher quit percentages, i.e. being in a preparation stage, being low tobacco dependent and having a delayed reward preference. Negative values are associated with lower quit percentages, i.e. being in a (pre-)contemplation stage, being dependent on tobacco and having an immediate reward preference.

A combination of three positive values leads to the ascending scheme, two positive values to the standard scheme and two or three negative values to the descending scheme (see table 1). Those

For peer review only - http://bmjopen.bmj.com/site/about/guidelines.xhtml

willing to pay a deposit are automatically advised the deposit-based scheme. The rationale behind this is that those with the lowest scores are likely to experience the most trouble quitting, and hence arguably benefit most from higher rewards in the beginning. Those with a higher score might rather benefit from incentives as a motivator to remain abstinent, and thus receive higher rewards towards the end of the study period. Importantly, participants are however free to choose the scheme of their preference when entering the trial regardless of the advice they received.

Table 1: Answer combinations and advised schemes

Degree of tobacco dependence*	Readiness-to-quit**	Present bias***	Willing to pay a deposit	Scheme advice
< 5	Ready to quit within the next 30 days	Delayed reward preference	No	Ascending
< 5	Ready to quit within the next 30 days	Present reward preference	No	Standard
< 5	Ready to quit in the next 6 months or not ready to quit	Delayed reward preference	No	Standard
< 5	Ready to quit in the next 6 months or not ready to quit	Present reward preference	No	Descending
≥ 5	Ready to quit within the next 30 days	Delayed reward preference	No	Standard
≥ 5	Ready to quit within the next 30 days	Present reward preference	No	Descending
≥ 5	Ready to quit in the next 6 months or not ready to quit	Delayed reward preference	No	Descending
≥ 5	Ready to quit in the next 6 months or not ready to quit	Present reward preference	No	Descending

For peer review only - http://bmjopen.bmj.com/site/about/guidelines.xhtml

Any answer	Any answer	Any answer	Yes	Deposit

** assessed via Fagerström score*

*** assessed via Prochaska state of change*

****assessed via Kirby discontinuity score*

As participants have freedom of choice over the incentive schemes, this is likely to result in a skewed distribution of participants over the schemes. However, this is not an issue in the current trial as the aim is to estimate the effect of personalised incentives compared to no incentives and not to estimate the discrete effects of the individual schemes. In fact, the current trial will provide information as to which schemes are most popular, how this relates to personal characteristics, and whether participants may be nudged into the scheme that is assumed to best fit their personal characteristics.

Outcomes

The primary outcome is CO-validated twelve month continuous abstinence from smoking (Russel's Standard, RS12).[23] The primary outcome is measured at T=12 months after the last group based training. However, participants jointly quit smoking at the third training session which is approximately one month prior to the last training session. This one month gap between the joint smoking attempt and the start of our measurement allows participants to have a setback in smoking cessation while they are still in the training. From the end of the training (T=0 months) onwards, all participants are expected to be smoke-free. Abstinence is assessed via self-reported abstinence and biochemically validated using expired air (CO) measurements. Self-reported abstinence assesses both point- and continuous abstinence. Point prevalence is assessed by seven-day abstinence at each follow-up point and prolonged abstinence via the total continuous abstinence period.[23] Self-reported abstinence is validated via assessment of expired air CO concentrations using a handheld monitor (PiCO™ Smokerlyzer, Bedfont Scientific Ltd., Kent, England). An exhaled-air CO reading of <10 ppm is considered indicative of abstinence.[23] CO quantification is a reliable and non-invasive method to ascertain recent smoking and the preferred method in populations using nicotine replacement therapy.[23] In case of disagreement between self-reported abstinence and CO reading, abstinence is defined by the latter.

Secondary outcomes are smoking abstinence directly after the group-based smoking cessation training (t=0), and after three months (t=3), after six months (t=6) and after fifteen months (t=15). The same methods and definitions to determine continuous abstinence are used for all outcomes.

Tertiary outcomes are the incremental cost-effectiveness ratio and the incremental cost utility ratio, where the first is calculated using the total costs per quitter and the latter by the costs and self-reported quality of life (EQ-5D).

Sample size

The CATCH trial, which employed similar paying schemes as the standard arm of the current trial, reported 41.1% abstinence at twelve months follow up in the intervention arm, versus 26.4% among participants who only attended the training sessions.[13] The PERSIST trial will be embedded in health care institutions, many of which are currently implementing smoke-free policies, which is expected to

For peer review only - http://bmjopen.bmj.com/site/about/guidelines.xhtml

be an extra external motivator for cessation. As such the validated abstinence percentages are expected to be slightly higher than in the CATCH trial, i.e. 45% and 30%, respectively. We expect that personalisation of the incentives will further increase effectiveness to approximately 50%. In order to detect this difference in validated sustained abstinence (i.e. 50% vs. 30%) with a power of 0.8 and an alpha of 0.05 and using 1:1 randomisation, a total of at least 186 participants is needed. Taking into account a 15% loss to follow-up due to unexpected employee turnover,[11,24] 220 participants are required. Further taking into account the potential maximum deviation from a 1:1 allocation ratio which may arise due to the stratified block randomisation procedure used, the minimum sample size required will increase in proportion to the number of different hospitals included in the trial. As the trial aims to estimate the effect of personalised incentives compared to no incentives and not to estimate the effect of each individual scheme, skewed allocation among the schemes is not an issue and is therefore not accounted for in the required sample size.

Recruitment

Recruitment takes place at participating health care institutions, starting with Erasmus MC. Potential participants are informed about the group-based smoking cessation training sessions through internal websites, flyers, information sessions provided by SineFuma or by referral through their company doctor. Potential participants receive a letter explaining the different incentive schemes and the research procedures. Upon enrolment, informed consent is required.

Allocation

Within participating institutions, participants will be individually randomised 1:1 to either the control or intervention arm. Permuted block randomisation with varying block sizes of four and six is used. There are two stratification factors: participating institution and readiness-to-quit (preparation or contemplation stage). A computer-generated allocation sequence was provided by ALEA randomisation service, in collaboration with the Erasmus MC Clinical Trial Centre. The block sizes will not be disclosed so as to ensure allocation concealment. ALEA will not release the randomisation result until the patient has been recruited into the trial and all baseline questionnaires have been completed. Participants are informed about their allocation by email. Inherent to the intervention and study design, participants and outcome assessors will not be blinded to allocation. Group session trainers will be blinded to allocation and data will be anonymised before analysis.

Collection methods

As described in the section 'Outcomes', self-reported abstinence will be validated using expired air CO concentration readings obtained via a handheld monitor. Participants will be invited to complete web-based questionnaires assessing self-reported quality of life (EQ-5DF),[25] smoking abstinence self-efficacy (SASEQ),[26] perceived stress (Perceived Stress Scale)[27] and self-reported smoking abstinence (point prevalence and continuous abstinence)[23] at each time point during follow-up. At baseline the following items will be collected: educational level following the International Standard Classification of Education (ISCED),[28] 0 (early childhood education) to 8 (doctoral or equivalent level), individual gross monthly income (<€1500, €1500-1900, €1900-2600, >€2600), age, Body Mass Index (BMI), gender and smoking behaviour (pack years, history of quit attempts, and smoking history). In order to provide a personalised advise the following questionnaires are also taken at baseline: nicotine dependency (Fagerström scale),[19] readiness-to-quit (Prochaska stages of change)[20] and present bias (Kirby discontinuity scale).[21,29] See table 2 for a schematic overview of data collection during the trial.

For peer review only - http://bmjopen.bmj.com/site/about/guidelines.xhtml

Participants will be sent reminders after five days for uncompleted questionnaires. A follow-up reminder will be sent two days later. If there are still open items, participants will be contacted by telephone two days after the last reminder and a follow-up phone call will be made another two days later.

Table 2: Schematic overview of data collection during the trial.

Questionnaire	Baseline	t = 0 months	t = 3 months	t = 6 months	t = 12 months *	t = 15 months
Demographic variables	X					
Fagerström scale	X					
Prochaska stage of change	X					
Kirby discontinuity scale	X					
Willingness to pay a deposit	X					
EQ-5DF**	X	X	X	X	X	X
SASEQ***	X	X	X	X	X	X
Perceived stress scale	X	X	X	X	X	X
Self-reported abstinence*** *	X	X	X	X	X	X
Evaluation training sessions		X				
Evaluation PERSIST						X

**t = 12 months is the primary endpoint*

*** EurQol Five Dimensions Health Questionnaire*

**** Smoking abstinence self-efficacy questionnaire*

***** The self-reported abstinence questionnaire is adjusted to each corresponding time moment, e.g. at t =3 the maximum period not smoked is three months etc.*

Data management

For peer review only

For peer review only - http://bmjopen.bmj.com/site/about/guidelines.xhtml

Informed consent will be provided digitally through LimeSurvey (LimeSurvey GmbH, Hamburg, Germany). All trial data will be entered electronically using GemsTracker (GemsTracker ©, Erasmus MC and *Equipe Zorgbedrijven*, Rotterdam, the Netherlands). Data are handled confidentially and stored anonymously. Where data need to be linked to an individual subject (e.g. linking longitudinal data), a subject identification code will be used. The code will not be based on patient initials and date of birth to preserve anonymity. The key to the code will be safeguarded by the investigator and kept separate from data files. All of the study essential documents will be retained and archived for 15 years after completion of the study. They will be stored securely and adequately protected from damage.

Statistical methods

The primary analysis compares continuous smoking abstinence rates at twelve months after the group-based training session in the intervention arm against the control arm, following an intention-to-treat analysis. Secondary continuous smoking abstinence at fifteen months is assessed to see if abstinence is sustained after the incentives stopped. All randomised participants will be included in the final analysis, those lost to follow up will be classified as smoking except for an unavoidable loss to follow up (e.g. untraceable contact information or death, following the Russel standard).[23] Generalised linear models with a logit link function will be used to assess the difference in the primary outcome between control and intervention arm at t =12 months and to account for clustering between hospitals.

Additionally, subgroup analyses will be performed to identify possible subgroup-specific effects of the intervention. Planned subgroup analyses are based on:

1. readiness-to-quit. Preparation stage versus contemplation stage;[10,20]
2. degree of tobacco dependence. Fagerström score below 5 versus Fagerström score of 5 or higher;[19]
3. present bias. Delayed versus present reward preference. Delayed reward preference = more than 50% larger delayed reward is chosen, or present reward preference = less than 50% larger delayed reward is chosen; [21,22]
4. income. Based on gross monthly income in €. Low income = below 1500 versus moderate/high income = above 1500.[30]

and

5. Educational level. Based on the International Classification of Education (ISCED).[28] Lower educated = ISCED 1-4 versus higher educated = ISCED 5-8.

Subgroup analysis are not included in the power analysis and therefore expected to be suggestive. For missing data on subgroup information, multiple imputation will be performed.

Patient and Public Involvement

Participants were not involved in designing the trial. Once the results are published participants will be informed about the publication by email. This email will be drafted suitable for a non-specialist audience. Public involvement will be sought through the Erasmus Initiative Smarter Choices for Better Health.

ETHICS AND DISSEMINATION

For peer review only - http://bmjopen.bmj.com/site/about/guidelines.xhtml

The study protocol has been assessed by the Erasmus MC Medical Ethics Committee (MEC-2019-0140). The committee decided that according to the Dutch Human Research Law (WMO) the protocol required no formal ethical approval. All participants will provide informed consent for participating in the study.

The results will be published in a peer-reviewed scientific journal. On completion of the trial, and after the publication of the results, data requests can be submitted to the corresponding author at Erasmus MC, Rotterdam, the Netherlands.

DISCUSSION

We present the protocol of the "PERSonalised Incentives for Supporting Tobacco cessation" (PERSIST) trial. The aim of the trial is to evaluate the effectiveness of personalised incentives in addition to group-based smoking cessation training on sustained smoking abstinence among hospital employees compared to group-based training alone. Personalisation entails that participants are advised and can choose from four different incentive schemes: standard, ascending, descending, and deposit-based.

Strengths and limitations

The PERSIST trial is unique in its approach to personalising incentives to promote smoking cessation. Strong links between personal characteristics and effectiveness of incentives to promote smoking cessation have been demonstrated previously, however, to our knowledge, the effectiveness of personalised incentives has not been studied before.[14] Another unique element of the PERSIST trial is that participants receive personalised advice for an incentive scheme, based on personal characteristics, yet are free to deviate from the advised scheme. Voluntary choice with a personalised default is a common tool in behavioural economics to nudge people towards the scheme that is plausibly in their best interest.[31] This feature is scientifically innovative in the context of smoking cessation programmes, and importantly let the treated individuals to choose the scheme they will be enrolled.

By focusing on supporting smoking cessation among hospital employees, the PERSIST trial is executed in a highly relevant setting as in light of the National Prevention Agreement all university hospitals have to be smoke free by 2020 and all general hospitals by 2025.[32] Finally, the PERSIST trial progresses from previous studies by extending follow-up beyond the last time point when incentives are provided to investigate if participants remain abstinent when incentives are no longer provided.

A limitation of the PERSIST trial is that participants from the intervention and control arm attend the same cessation support training sessions due to practical reasons, as slow inclusion rates otherwise would have caused long waiting periods between signing up and starting the training. Participants in the control arm might perceive this negatively and potentially drop out, causing attrition bias. Another consequence could be spill-over effects;[33] sharing of vouchers between control and intervention arm, leading to a double underestimation of treatment effectiveness. To avoid this, participants are informed about their status as early as possible to make sure they have time to accept their status. A third arm consisting of non-personalised incentives could have provided additional potentially valuable information on the effectiveness of personalised incentives versus incentives provided according to a fixed scheme. However, this option would have required unfeasibly large sample size.

Availability of data and material

Data will become available upon completion of the study

Declarations

For peer review only - http://bmjopen.bmj.com/site/about/guidelines.xhtml

- Consent for publication
  Not applicable, no personal or identifying data will be published.
- Access to the data
  Data will become available upon completion of the study, which is expected to be beginning of 2023.
- Amendments
  Important protocol modifications will be communicated to the METC and trial participants
- Competing interests
  The authors declare that they have no competing interests
- Funding
  This study is funded by the Erasmus Initiative Smarter Choices for Better Health and the Erasmus Trustfonds. JVB is funded by a personal fellowship from the Netherlands Lung Foundation.
- Author Contributions
  JVB, FvL and HvK wrote the initial version of the programme grant request on which this protocol was based. NWB drafted the manuscript and runs the day-to-day study. DTOC did the sample size calculations, advised on the randomisation method and drafted the allocation algorithm and MKR advised on the statistical analysis. The paper was revised and edited by NWB, HvK, MKR, DTOC, AB, FvL and JVB and all authors approved the final manuscript.
- Progress of the trial
  Currently the trial is ongoing and open for inclusion.

For peer review only

Abbreviations

CI: Confidence interval

CO: Carbon monoxide

Erasmus MC: Erasmus University Medical Centre

PERSIST: Personalised Incentives for Supporting Tobacco Cessation among Health care Employees, title of the project

Ppm: Parts per million

RR: Relative Risk

REFERENCES

1. Forouzanfar MH, Afshin A, Alexander LT, et al. Global, regional, and national comparative risk assessment of 79 behavioural, environmental and occupational, and metabolic risks or clusters of risks, 1990–2015: a systematic analysis for the Global Burden of Disease Study 2015. *The Lancet* 2016;**388**(10053):1659-724.
2. Nayak P, Pechacek TF, Slovic P, et al. Regretting Ever Starting to Smoke: Results from a 2014 National Survey. *Int J Environ Res Public Health* 2017;**14**(4):12.

For peer review only - http://bmjopen.bmj.com/site/about/guidelines.xhtml

3. Netherlands S. Leefstijl en (preventief) gezondheidsonderzoek; persoonskenmerken. 19-03-2019 ed: CBS, 2019.
4. Stead LF, Perera R, Bullen C, et al. Nicotine replacement therapy for smoking cessation. *Cochrane Database Syst Rev* 2012(11).
5. Lancaster T, Stead LF. Individual behavioural counselling for smoking cessation. *Cochrane Database Syst Rev* 2017(3).
6. Stead LF, Carroll AJ, Lancaster T. Group behaviour therapy programmes for smoking cessation. *Cochrane Database Syst Rev* 2017(3).
7. Notley C, Gentry S, Livingstone-Banks J, et al. Incentives for smoking cessation. *Cochrane Database Syst Rev* 2019(7).
8. Odum AL. Delay discounting: I'm a k, you're a k. *J Exp Anal Behav* 2011;**96**(3):427-39.
9. O'Donoghue T, Rabin M. Doing it now or later. *Am Econ Rev* 1999;**89**(1):103-24.
10. Halpern SD, French B, Small DS, et al. Randomized trial of four financial-incentive programs for smoking cessation. *N Engl J Med* 2015;**372**(22):2108-17.
11. Volpp KG, Troxel AB, Pauly MV, et al. A randomized, controlled trial of financial incentives for smoking cessation. *N Engl J Med* 2009;**360**(7):699-709.
12. Halpern SD, Harhay MO, Saulsgiver K, et al. A Pragmatic Trial of E-Cigarettes, Incentives, and Drugs for Smoking Cessation. *N Engl J Med* 2018;**378**(24):2302-10.
13. van den Brand FA, Nagelhout GE, Winkens B, et al. Effect of a workplace-based group training programme combined with financial incentives on smoking cessation: a cluster-randomised controlled trial. *Lancet Public Health* 2018;**3**(11):e536-e44.
14. Halpern SD, French B, Small DS, et al. Heterogeneity in the Effects of Reward- and Deposit-based Financial Incentives on Smoking Cessation. *Am J Respir Crit Care Med* 2016;**194**(8):981-88.
15. Chan A-W, Tetzlaff JM, Altman DG, et al. SPIRIT 2013: new guidance for content of clinical trial protocols. *Lancet* 2013;**381**(9861):91-92.
16. Milieu RvVe. Loket gezond leven *RIVM* 2019 [Available from: https://interventies.loketgezondleven.nl/leefstijlinterventies/interventies-zoeken/1400743.
17. Gneezy U, Meier S, Rey-Biel P. When and why incentives (don't) work to modify behavior. *J Econ Perspect* 2011;**25**(4):191-210.
18. van den Brand FA, Nagelhout GE, Winkens B, et al. Effect of a workplace-based group training programme combined with financial incentives on smoking cessation: a cluster-randomised controlled trial. *The Lancet Public Health* 2018;**3**(11):e536-e44.
19. Heatherton TF, Kozlowski LT, Frecker RC, et al. The Fagerstrom Test for Nicotine Dependence: a revision of the Fagerstrom Tolerance Questionnaire. *Br J Addict* 1991;**86**(9):1119-27.
20. Prochaska JO, Velicer WF, DiClemente CC, et al. Measuring processes of change: applications to the cessation of smoking. *J Consult Clin Psychol* 1988;**56**(4):520-8.
21. Kirby KN. Bidding on the future: Evidence against normative discounting of delayed rewards. *J Exp Psychol Gen* 1997;**126**(1):54-70.
22. Kaplan BA, Amlung M, Reed DD, et al. Automating Scoring of Delay Discounting for the 21- and 27-Item Monetary Choice Questionnaires. *Behav Analyst* 2016;**293-304**(2):12.
23. West R, Hajek P, Stead L, et al. Outcome criteria in smoking cessation trials: proposal for a common standard. *Addiction* 2005;**100**(3):299-303.
24. van den Brand FA, Nagelhout GE, Winkens B, et al. Effect of a workplace-based group training programme combined with financial incentives on smoking cessation: a cluster-randomised controlled trial. *Lancet Public Health* 2018;**3**(11):9.
25. Herdman M, Gudex C, Lloyd A, et al. Development and preliminary testing of the new five-level version of EQ-5D (EQ-5D-5L). *Qual Life Res* 2011;**20**(10):1727-36.
26. Spek V, Lemmens F, Chatrou M, et al. Development of a smoking abstinence self-efficacy questionnaire. *Int J Behav Med* 2013;**20**(3):444-9.
27. Cohen S, Kamarck T, Mermelstein R. A global measure of perceived stress. *J Health Soc Behav* 1983;**24**(4):385-96.

For peer review only - http://bmjopen.bmj.com/site/about/guidelines.xhtml

28. Unesco Institute for Statistics. International standard classification of education: ISCED 2011: UNESCO Institute for Statistics Montreal, 2012.
29. Gray JC, Amlung MT, Palmer AA, et al. Syntax for calculation of discounting indices from the monetary choice questionnaire and probability discounting questionnaire. *J Exp Anal Behav* 2016;**106**(2):156-63.
30. Netherlands S. Inkomensgroepen: Particuliere huishoudens: *Statistics Netherlands*; 2014 [updated 15-04-2018. Available from: https://statline.cbs.nl/Statweb/publication/?DM=SLNL&PA=71013ned&D1=0-2&D2=1-2&D3=11-14&D4=44-62&D5=l&HDR=G4,G1,T&STB=G3,G2&VW=L.
31. Schlag P. Nudge, choice architecture, and libertarian paternalism: HeinOnline, 2009.
32. The National Prevention Agreement In: Ministry of Health WaS, ed. The Hague 2019.
33. Angelucci M, Maro VD. Program evaluation and spillover effects: The World Bank, 2015.

For peer review only

For peer review only - http://bmjopen.bmj.com/site/about/guidelines.xhtml

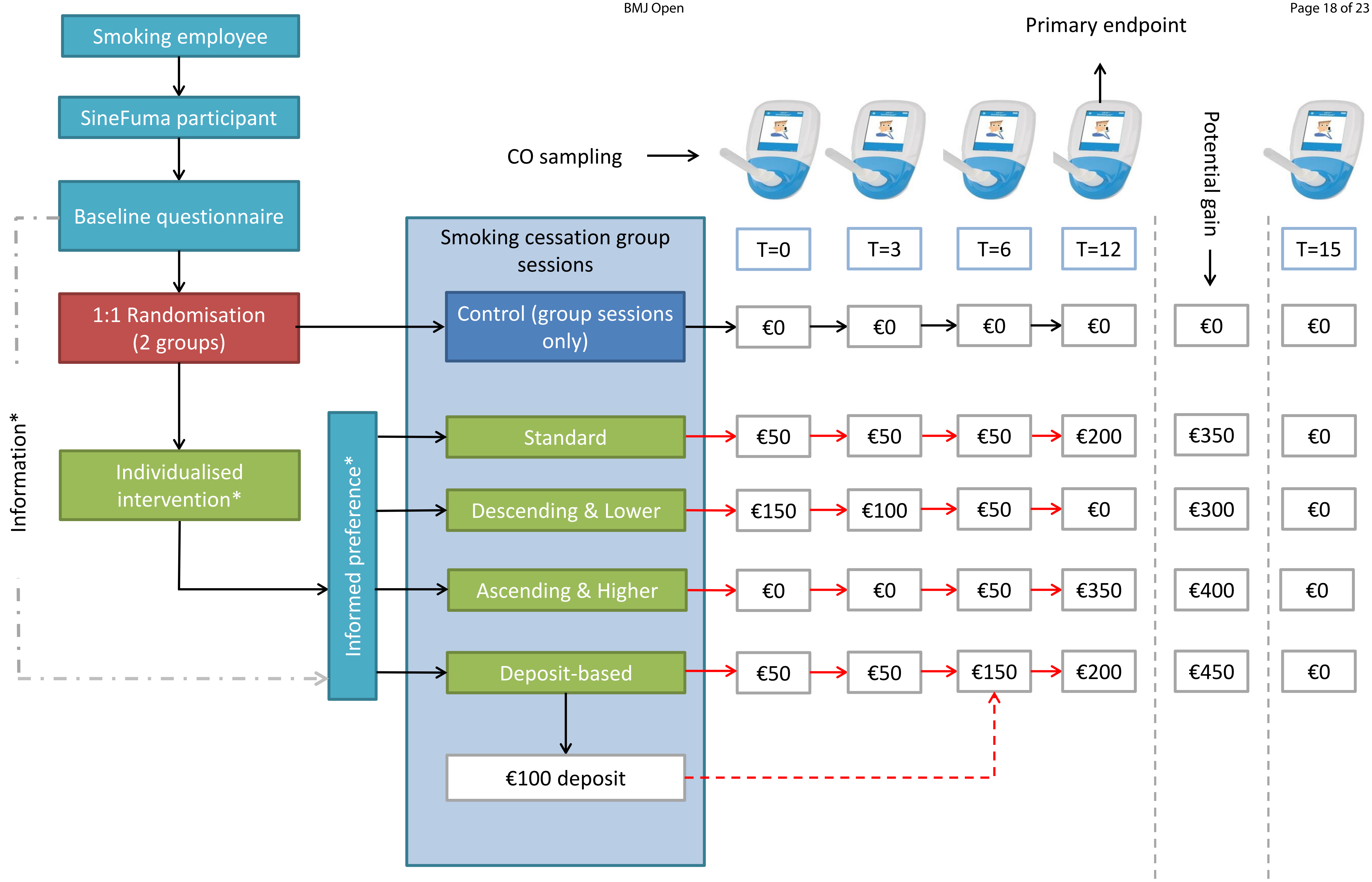

*Participants are provided with an informed choice regarding the individualised incentive scheme based on: 1. degree of tobacco dependence, 2. Readiness to quit and 3. Present-bias.
Note: red arrows are conditional on sustained biochemically validated smoking cessation

PERSIST – SPIRIT CHECKLIST

Section/item	ItemNo	Description	Page number
**Administrative information**			
Title	1	Descriptive title identifying the study design, population, interventions, and, if applicable, trial acronym	1
Trial registration	2a	Trial identifier and registry name. If not yet registered, name of intended registry	2
	2b	All items from the World Health Organization Trial Registration Data Set	NA
Protocol version	3	Date and version identifier	
Funding	4	Sources and types of financial, material, and other support	14
Roles and responsibilities	5a	Names, affiliations, and roles of protocol contributors	1
	5b	Name and contact information for the trial sponsor	NA
	5c	Role of study sponsor and funders, if any, in study design; collection, management, analysis, and interpretation of data; writing of the report; and the decision to submit the report for publication, including whether they will have ultimate authority over any of these activities	NA
	5d	Composition, roles, and responsibilities of the coordinating centre, steering committee, endpoint adjudication committee, data management team, and other individuals or groups overseeing the trial, if applicable (see Item 21a for data monitoring committee)	NA
**Introduction**			
Background and rationale	6a	Description of research question and justification for undertaking the trial, including summary of relevant studies (published and unpublished) examining benefits and harms for each intervention	4
	6b	Explanation for choice of comparators	NA
Objectives	7	Specific objectives or hypotheses	5

For peer review only - http://bmjopen.bmj.com/site/about/guidelines.xhtml

Trial design	8	Description of trial design including type of trial (eg, parallel group, crossover, factorial, single group), allocation ratio, and framework (eg, superiority, equivalence, noninferiority, exploratory)	5
**Methods: Participants, interventions, and outcomes**			
Study setting	9	Description of study settings (eg, community clinic, academic hospital) and list of countries where data will be collected. Reference to where list of study sites can be obtained	5
Eligibility criteria	10	Inclusion and exclusion criteria for participants. If applicable, eligibility criteria for study centres and individuals who will perform the interventions (eg, surgeons, psychotherapists)	5
Interventions	11a	Interventions for each group with sufficient detail to allow replication, including how and when they will be administered	5
	11b	Criteria for discontinuing or modifying allocated interventions for a given trial participant (eg, drug dose change in response to harms, participant request, or improving/worsening disease)	NA
	11c	Strategies to improve adherence to intervention protocols, and any procedures for monitoring adherence (eg, drug tablet return, laboratory tests)	NA
	11d	Relevant concomitant care and interventions that are permitted or prohibited during the trial	NA
Outcomes	12	Primary, secondary, and other outcomes, including the specific measurement variable (eg, systolic blood pressure), analysis metric (eg, change from baseline, final value, time to event), method of aggregation (eg, median, proportion), and time point for each outcome. Explanation of the clinical relevance of chosen efficacy and harm outcomes is strongly recommended	9
Participant timeline	13	Time schedule of enrolment, interventions (including any run-ins and washouts), assessments, and visits for participants. A schematic diagram is highly recommended (see Figure)	6

For peer review only - http://bmjopen.bmj.com/site/about/guidelines.xhtml

Sample size	14	Estimated number of participants needed to achieve study objectives and how it was determined, including clinical and statistical assumptions supporting any sample size calculations	9
Recruitment	15	Strategies for achieving adequate participant enrolment to reach target sample size	10
**Methods: Assignment of interventions (for controlled trials)**			
Allocation:			10
Sequence generation	16a	Method of generating the allocation sequence (eg, computer-generated random numbers), and list of any factors for stratification. To reduce predictability of a random sequence, details of any planned restriction (eg, blocking) should be provided in a separate document that is unavailable to those who enrol participants or assign interventions	10
Allocation concealment mechanism	16b	Mechanism of implementing the allocation sequence (eg, central telephone; sequentially numbered, opaque, sealed envelopes), describing any steps to conceal the sequence until interventions are assigned	10
Implementation	16c	Who will generate the allocation sequence, who will enrol participants, and who will assign participants to interventions	10
Blinding (masking)	17a	Who will be blinded after assignment to interventions (eg, trial participants, care providers, outcome assessors, data analysts), and how	10
	17b	If blinded, circumstances under which unblinding is permissible, and procedure for revealing a participant's allocated intervention during the trial	NA
**Methods: Data collection, management, and analysis**			

Data collection methods	18a	Plans for assessment and collection of outcome, baseline, and other trial data, including any related processes to promote data quality (eg, duplicate measurements, training of assessors) and a description of study instruments (eg, questionnaires, laboratory tests) along with their reliability and validity, if known. Reference to where data collection forms can be found, if not in the protocol	10
	18b	Plans to promote participant retention and complete follow-up, including list of any outcome data to be collected for participants who discontinue or deviate from intervention protocols	10
Data management	19	Plans for data entry, coding, security, and storage, including any related processes to promote data quality (eg, double data entry; range checks for data values). Reference to where details of data management procedures can be found, if not in the protocol	11
Statistical methods	20a	Statistical methods for analysing primary and secondary outcomes. Reference to where other details of the statistical analysis plan can be found, if not in the protocol	12
	20b	Methods for any additional analyses (eg, subgroup and adjusted analyses)	12
	20c	Definition of analysis population relating to protocol non-adherence (eg, as randomised analysis), and any statistical methods to handle missing data (eg, multiple imputation)	12
**Methods: Monitoring**			
Data monitoring	21a	Composition of data monitoring committee (DMC); summary of its role and reporting structure; statement of whether it is independent from the sponsor and competing interests; and reference to where further details about its charter can be found, if not in the protocol. Alternatively, an explanation of why a DMC is not needed	12
	21b	Description of any interim analyses and stopping guidelines, including who will have access to these interim results and make the final decision to terminate the trial	NA

Harms	22	Plans for collecting, assessing, reporting, and managing solicited and spontaneously reported adverse events and other unintended effects of trial interventions or trial conduct	NA
Auditing	23	Frequency and procedures for auditing trial conduct, if any, and whether the process will be independent from investigators and the sponsor	NA
**Ethics and dissemination**			
Research ethics approval	24	Plans for seeking research ethics committee/institutional review board (REC/IRB) approval	12
Protocol amendments	25	Plans for communicating important protocol modifications (eg, changes to eligibility criteria, outcomes, analyses) to relevant parties (eg, investigators, REC/IRBs, trial participants, trial registries, journals, regulators)	14
Consent or assent	26a	Who will obtain informed consent or assent from potential trial participants or authorised surrogates, and how (see Item 32)	11
	26b	Additional consent provisions for collection and use of participant data and biological specimens in ancillary studies, if applicable	NA
Confidentiality	27	How personal information about potential and enrolled participants will be collected, shared, and maintained in order to protect confidentiality before, during, and after the trial	11
Declaration of interests	28	Financial and other competing interests for principal investigators for the overall trial and each study site	14
Access to data	29	Statement of who will have access to the final trial dataset, and disclosure of contractual agreements that limit such access for investigators	14
Ancillary and post-trial care	30	Provisions, if any, for ancillary and post-trial care, and for compensation to those who suffer harm from trial participation	NA

For peer review only

Dissemination policy	31a	Plans for investigators and sponsor to communicate trial results to participants, healthcare professionals, the public, and other relevant groups (eg, via publication, reporting in results databases, or other data sharing arrangements), including any publication restrictions	12
	31b	Authorship eligibility guidelines and any intended use of professional writers	14
	31c	Plans, if any, for granting public access to the full protocol, participant-level dataset, and statistical code	NA
**Appendices**			
Informed consent materials	32	Model consent form and other related documentation given to participants and authorised surrogates	17
Biological specimens	33	Plans for collection, laboratory evaluation, and storage of biological specimens for genetic or molecular analysis in the current trial and for future use in ancillary studies, if applicable	NA

For peer review only

For peer review only - http://bmjopen.bmj.com/site/about/guidelines.xhtml
